# Multi-Parameter Modulation of Dirac Plasmons in Germanene via Doping and Strain: A DFT Insight

**DOI:** 10.3390/ma18214824

**Published:** 2025-10-22

**Authors:** Pengfei Li, Lijun Han, Lin Zhang, Ningju Hui

**Affiliations:** 1 Department of Applied Physics, Xi’an University of Technology, Xi’an 710048, China; 2 Shaanxi University Key Laboratory of Photonic Power Devices and Discharge Regulation, Xi’an University of Technology, Xi’an 710048, China

**Keywords:** first principles, germanene, electronic excitations, loss spectra, plasmon

## Abstract

Based on first-principles calculations and linear-response time-dependent density functional theory within the random phase approximation (LR-TDDFT-RPA), this work systematically investigates the modulation of Dirac plasmons in germanene via carrier doping, biaxial strain, and substrate effects. The results demonstrate that carrier doping induces highly tunable Dirac plasmons whose excitation energy follows the ω ∝ n^1/4^ scaling relation, leading to a sublinear increase with doping concentration. Furthermore, biaxial strain effectively modulates the Fermi velocity, and the established ω ∝ √V_F_ relationship directly explains the observed linear tuning of plasmon energy with strain. More importantly, the combined modulation of carrier density and strain enables a significantly broader plasmon energy range (0.16–0.61 eV) than achievable through individual parameter control. When supported on hBN substrates, germanene maintains the characteristic √q plasmon dispersion despite band hybridization and a redshift in energy, a behavior well explained by the 2D free electron gas model. This study provides important theoretical insights into the multi-parameter control of Dirac plasmons and supports the design of germanene-based tunable nanophotonic devices.

## 1. Introduction

Germanene, a two-dimensional honeycomb lattice composed of germanium atoms from group-IV, has become a prominent research topic in condensed matter physics and nanoelectronics due to its graphene-like Dirac cone band structure and notable structural buckling [1]. Unlike perfectly flat graphene, the larger ionic radius of germanium and strong relativistic effects lead to mixed sp^2^–sp^3^ hybridization, resulting in a low-buckled geometry. This structural distortion breaks sublattice mirror symmetry and significantly enhances spin–orbit coupling (SOC). Theoretical calculations predict that germanene may be a quantum spin Hall insulator with a sizable spin–orbit coupling gap [2,3], though its topological insulating state has not yet been experimentally confirmed. Due to strong interlayer interactions in bulk germanium, mechanical exfoliation of germanene remains challenging. Thus, current synthesis methods mainly depend on epitaxial growth on metal substrates such as Pt(111) [4], Au(111) [5], or Cu(111) [6]. Multilayer Bernal stacking can effectively screen substrate coupling, restoring Dirac electronic states and showing promise for ultrafast nonlinear optical modulation [7,8]. Moreover, surface functionalization strategies—including hydrogenation and halogenation—greatly expand germanene’s property portfolio: hydrogenated germanene exhibits superconducting transitions under high pressure [9], while halogenation can introduce long-range ferromagnetic order, offering a new platform for realizing the quantum anomalous Hall effect [10].

As light–matter interactions approach the deep-subwavelength scale, plasmonics has emerged as a key framework for controlling nanoscale optical fields, enabling advances in super-resolution imaging [11], on-chip quantum light sources [12], and optoelectronic integration [13]. However, conventional noble metal plasmons are inherently limited by high Ohmic losses, creating a fundamental trade-off between strong field confinement and energy dissipation. Two-dimensional (2D) materials, with extreme in-plane confinement due to atomic-scale thickness, naturally suppress out-of-plane radiation losses, offering a promising path to overcome this limitation [14]. The unique properties of Dirac plasmons in two-dimensional materials—such as their dynamic tunability, strong field confinement, and low optical losses—propel them beyond fundamental interest and towards transformative applications in nano-optoelectronics. Unlike their noble metal counterparts, the resonant response of these collective excitations can be actively modulated post-fabrication via electrical gating, presenting a pivotal advantage for the development of reconfigurable metamaterials and high-speed, ultra-compact optical modulators [15]. Furthermore, their capacity to concentrate light into deep-subwavelength volumes, coupled with intrinsic resonances that span the mid-infrared molecular fingerprint region, establishes them as an unparalleled platform for surface-enhanced infrared absorption (SEIRA) spectroscopy, enabling ultrasensitive and label-free biochemical detection [16]. The van der Waals nature of the host materials further ensures facile integration with diverse photonic architectures, opening avenues for engineering novel heterostructures where light–matter interactions can be tailored at the quantum level, thereby paving the way for advanced on-chip devices such as nanoscale lasers and quantum information processing systems [17]. Graphene, as the most widely studied 2D material, has had its Dirac plasmons thoroughly examined through both theoretical [18,19,20,21] and experimental [22,23,24,25] studies. This progress naturally inspires interest in germanene, another group-IV Dirac material. Although there have been occasional studies [26,27,28,29] on germanene plasmons in recent years, a systematic exploration of how doping and strain affect its Dirac plasmons is still lacking. In this work, using first-principles linear-response time-dependent density functional theory (LR-TDDFT) [30,31] calculations, we quantitatively investigate the influence of doping and strain on the Dirac plasmons in germanene and clarify the underlying physical mechanisms. This work provides several key advances in understanding the plasmon properties of germanene. We present a comprehensive theoretical and numerical analysis that quantitatively deciphers the relationship between Dirac plasmon energy and key parameters, including carrier concentration and Fermi velocity. Beyond individual tuning mechanisms, we further explore the cooperative effects of carrier doping and biaxial strain, demonstrating significantly enhanced modulation capability. Additionally, the impact of substrate interaction on plasmon dispersion is systematically investigated, confirming the persistence of fundamental plasmon characteristics in practical heterostructure environments.

The remainder of this paper is structured as follows: Section 2 outlines the workflow of the linear-response time-dependent density functional theory (LR-TDDFT) method and details the key parameters used for ground-state and LR-TDDFT calculations. Section 3 presents and discusses the ab initio results and theoretical analysis. Finally, Section 4 summarizes the conclusions and offers perspectives for future research.

## 2. Methods and Computational Details

This study employed first-principles density functional theory (DFT) to investigate the plasmon properties of germanene. The computational approach integrated electronic structure determination with subsequent analysis of plasmonic responses, utilizing the ABACUS 2.0.1 (Atomic-Orbital-Based Ab Initio Computation at USTC) software package [32,33].

Norm-conserving SG15 optimized multiple projector pseudopotentials [34] were used to describe ion–electron interactions, and the exchange-correlation functional was treated within the generalized gradient approximation (GGA) using the Perdew–Burke–Ernzerhof (PBE) parameterization [35]. The Kohn–Sham wavefunctions were expanded in a plane-wave basis with a kinetic energy cutoff of 50 Ry. Electronic convergence was achieved with a charge density tolerance of 1 × 10^−9^ eV, and structural relaxations were conducted using convergence thresholds of 0.1 kbar for stress and 0.01 eV/Å for atomic forces. For germanene, a 20 Å vacuum layer was applied along the *z*-axis to mitigate inter-layer coupling. A 200 × 200 × 1 k-point mesh was adopted for Brillouin zone sampling. The computed band energies and wavefunctions served as essential inputs for the subsequent plasmon analysis.

Furthermore, we employ linear-response time-dependent density functional theory within the random phase approximation (LR-TDDFT-RPA) to investigate the plasmonic properties of germanene [36,37,38,39]. The random phase approximation (RPA) [40,41] provides a fundamental theoretical framework for understanding the origin of plasmons—collective excitations arising from long-range Coulomb interactions in the electron gas. This approach simplifies the many-electron problem by treating the motion of each electron in a dynamic mean field generated by all other electrons, thereby effectively capturing collective oscillation modes in the electron gas. Although it neglects complex short-range correlations, the RPA accurately retains the long-range Coulomb interactions essential for driving plasmons. As a result, it enables first-principles predictions of plasmon existence and their characteristic frequencies, establishing RPA as a foundational and highly successful theoretical framework for describing plasmons and other collective excitations in metallic systems. Within the RPA framework, the dielectric function is given by ε(q, ω) = 1 − v(q)χ^0^(q, ω), where v(q) is the Coulomb potential and χ^0^(q, ω) is the non-interacting response function. Theoretically, plasmon modes correspond to the condition Re[ε(q, ω)] = 0 with a small Im[ε(q, ω)]. In our calculations, however, we identify plasmons from the peak positions in the electron energy loss spectrum (EELS) [42], where the spectral intensity is proportional to Im[−1/ε(q, ω)]. These two approaches are fundamentally equivalent and complementary, differing mainly in their physical interpretations and emphases. In summary, the RPA-based LR-TDDFT method, as implemented in the ABACUS package [43,44], accurately describes the plasmon behavior in germanene and provides a reliable theoretical foundation for analyzing the effects of doping, strain, and substrates on Dirac plasmons.

The computational procedure for obtaining the EELS spectrum encompassed the following steps:

(1) Calculation of the imaginary component χ^s^ of the non-interacting Kohn–Sham response function χ^0^ [45,46]:

(1) $$\begin{array}{l} \chi_{G,G\prime }^{s}(q,\omega )=\frac{1}{\Omega }\sum_{n,n\prime }\left(f_{n,k}-f_{n\prime ,k+q}\right)\delta (\omega +\varepsilon_{n,k}-\varepsilon_{n\prime ,k+q})\\ \times \left\langle n,k\right.|e^{-i(q+G)r}|\left.n\prime ,k+q\right\rangle \left\langle n\prime ,k+q\right.|e^{-i(q+G\prime )r\prime }|\left.n,k\right\rangle . \end{array}$$

Here, Ω is the Born–von Karman supercell volume, ω denotes the frequency, k is the wave vector within the first Brillouin zone, q represents the momentum transfer, and G, G′ are reciprocal lattice vectors. The terms f_n,k_, ε_n,k_, and |n,k⟩ correspond to Fermi occupation numbers, Kohn–Sham eigenvalues, and eigenvectors, respectively, with n and n′ indexing occupied and unoccupied states.

(2) Evaluation of the full non-interacting response function χ^0^ via the Hilbert transform:

(2) $$\chi_{G,G\prime }^{0}(q,\omega )=\int_{0}^{\infty }d\omega \prime (\frac{1}{\omega -\omega \prime +i\eta }-\frac{1}{\omega +\omega \prime +i\eta })\chi_{G,G\prime }^{s}(q,\omega \prime ).$$

where η is a smearing parameter that ensures numerical convergence.

(3) Construction of the interacting response function χ using the Dyson equation [47]:

(3) $$\chi_{G,G\prime }(q,\omega )=\chi_{G,G\prime }^{0}(q,\omega )+\sum_{G_{1},G_{2}}\chi_{G,G_{1}}^{0}(q,\omega )K_{G_{1},G_{2}}(q,\omega )\chi_{G_{2},G}(q,\omega ).$$

Within the random phase approximation (RPA), the kernel K contains only the Coulomb term. A truncated Coulomb potential [48] was applied to eliminate spurious interactions between periodic images in the two-dimensional system:

(4) $$K_{G_{1},G_{2}}(\bar{q},\omega )=\frac{4\pi \delta_{G_{1},G_{2}}}{{\left|\bar{q}+{\bar{G}}_{1}\right|}^{2}}[1-{\left(-1\right)}^{n_{z}}e^{-|\bar{q}+{\bar{G}}_{1}|\frac{L_{z}}{2}}].$$

where G = ($\bar{G}$, $G_{z}$), q = ($\bar{q}$, 0), with $\bar{G}$ and $\bar{q}$ denoting 2D in-plane reciprocal lattice vectors and Bloch momenta. The parameter L_z_ is the lattice vector length along z, and n_z_ = G_z_L_z_/2π (an integer).

(4) Computation of the inverse dielectric function:

(5) $$\varepsilon_{G,G\prime }^{-1}=\delta_{G,G\prime }+{\left(v\chi \right)}_{G,G\prime .}$$

where *v* denotes the 2D Coulomb potential.

(5) Identification of plasmon excitations as peaks in the energy loss spectrum:

(6) $$E_{Loss}\propto -Im\{\varepsilon_{G=0,G\prime =0}^{-1}\}$$

Only the head component (G = G′ = 0) was evaluated, corresponding to the macroscopic dielectric response. 

For all LR-TDDFT-RPA calculations, a broadening parameter η = 0.01 Ry was used; we have demonstrated the rationality of this parameter value through convergence tests in Appendix A, and the response matrices were expanded with 50 reciprocal G-vectors.

## 3. Results and Discussions

### 3.1. The Overall Characteristics of Germanene Plasmons

**Figure 1:**
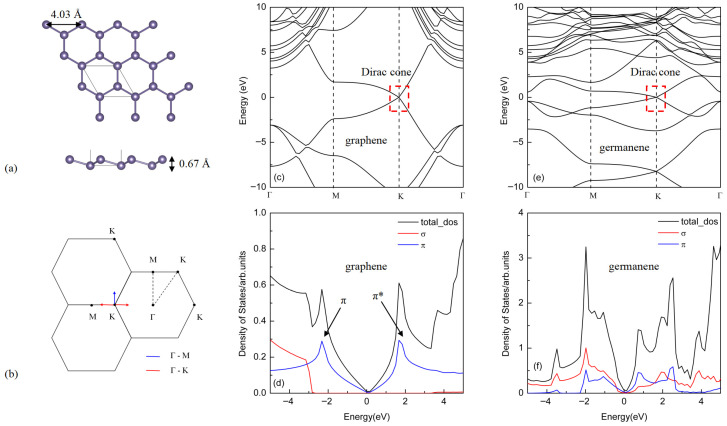
Geometric and electronic structures of graphene and germanene: (**a**) Top and side views of germanene, with a lattice constant of 4.03 Å and a buckling parameter of 0.67 Å; (**b**) reciprocal space structure of graphene/germanene, showing high-symmetry points and directions. The blue and red lines represent the Γ–M and Γ–K directions, respectively; (**c**) band structure of graphene; (**d**) the total and orbital-projected density of states of graphene: total (black), π-band (blue), σ-band (red); (**e**) band structure of germanene; (**f**) the total and orbital-projected density of states of germanene: total (black), π-band (blue), σ-band (red).

As illustrated in Figure 1a, germanene exhibits a distinct buckled lattice structure. This phenomenon originates from the larger atomic radius of germanium compared to carbon; the elongated Ge-Ge bonds result in substantially weakened π-orbital overlap, rendering it insufficient to maintain a planar graphene-like configuration. To achieve thermodynamic stability, the lattice undergoes buckling distortion—a structural adaptation that not only reduces system energy but also mitigates interatomic electrostatic repulsion. Concurrently, this structural rearrangement induces a hybridization transition from purely sp^2^ to partial^3^ character. It has been substantiated by both theoretical calculations [1] and experimental validation [5]. After structural relaxation, the optimized lattice constant and buckling height of germanene are 4.03 Å and 0.67 Å, respectively, consistent with existing experimental results [49]. Figure 1b illustrates the reciprocal space structure corresponding to the two-dimensional hexagonal honeycomb lattice shared by graphene and germanene, including the high-symmetry points Γ (center of the hexagon), M (midpoint of the edge), and K (vertex). Figure 1c–f present a comparative analysis of the band structures, total density of states (TDOS), and orbital-projected density of states (PDOS) of graphene and germanene. It can be observed that the two materials share similarities but also exhibit notable differences in their electronic structures. A key common feature is the presence of massless Dirac fermion behavior near the K point (Dirac point) in the Brillouin zone, where the valence and conduction bands show linear dispersion, forming the characteristic Dirac cone structure (highlighted by red boxes in Figure 1c,e). The linear band dispersion near the Dirac point in germanene is a direct consequence of its low-energy electrons behaving as massless Dirac fermions, a hallmark of its ideal two-dimensional hexagonal honeycomb lattice with two equivalent sublattices. This linearity can be derived from a tight-binding model considering nearest-neighbor hopping, where the Hamiltonian yields a linear energy-momentum relationship upon expansion around the K point. This characteristic electronic structure is also conclusively confirmed by the previous literature [1] by first-principles calculations. The main differences are as follows: graphene exhibits a sharp π/π* density of states peak around ±2 eV relative to the Fermi level, formed by p_z_ orbitals and corresponding to a saddle point near the M point in its band structure. In germanene, however, significant lattice buckling breaks the degeneracy at this location, resulting in a broadened and split π/π* peak. Consequently, the density of states in germanene does not show a sharp and tall van Hove singularity (VHS) peak as in graphene; instead, it displays a broader and smoother distribution. Furthermore, unlike the σ-bands in graphene—formed by s, p_x_, and p_y_ orbitals, which lie far from the Fermi level—the σ-bands in germanene shift closer to the Fermi level and overlap with the π-bands. This phenomenon further increases the complexity of the electronic structure of germanene.

**Figure 2:**
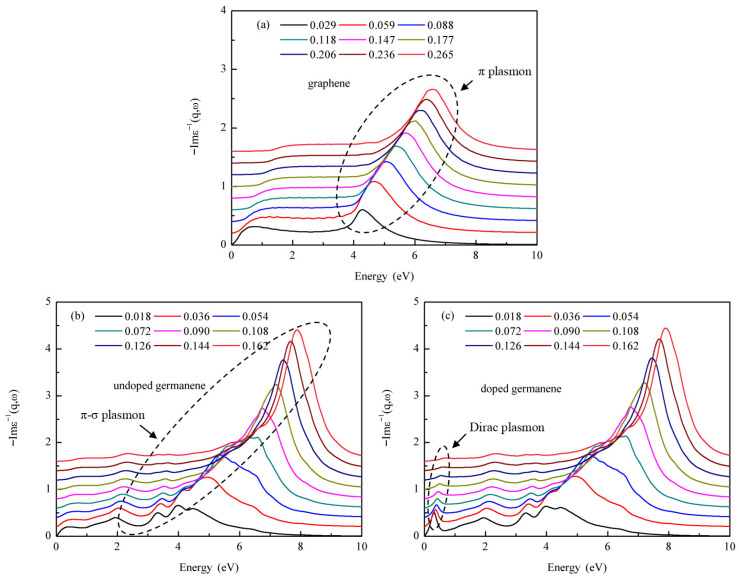
Momentum-resolved electron energy loss spectroscopy (EELS) of graphene and germanene: (**a**) EELS spectrum of undoped graphene along the Γ–M direction, with momentum transfer q ranging from 0.029 to 0.256 Å^−1^. The π plasmon is clearly visible; (**b**) EELS spectrum of undoped germanene along Γ–M, showing a combined π–σ plasmon across q = 0.018 − 0.162 Å^−1^; (**c**) EELS spectrum of doped germanene (*n* = 0.05 electrons/unitcell) along Γ–M, exhibiting a pronounced Dirac plasmon.

Figure 2a,b present the electron energy loss spectroscopy (EELS) of graphene and germanene along the Γ–M direction, respectively. Owing to its relatively simple electronic structure near the Fermi level, graphene exhibits a single prominent peak in the energy range of 4–8 eV. This peak originates from electronic transitions between the π and π* peaks in the density of states and is identified as the π plasmon mode (indicated by the dashed circle in Figure 2a). In contrast, germanene displays multiple overlapping peaks within a similar energy range (2–8 eV), reflecting various interband transitions between π and σ states in its density of states. This complex spectral feature is thus classified as a mixed π–σ plasmon mode (enclosed by the dashed circle in Figure 2b). Upon carrier doping in germanene, a new plasmon peak emerges below 1 eV (marked by the dashed circle in Figure 2c), in addition to the retained π–σ plasmon characteristics at higher energies. This low-energy mode arises from the collective oscillations of doped carriers near the Dirac point and is referred to as the Dirac plasmon. These results are consistent with and validate the findings reported in our previous work [43]. Among these plasmon modes, the π–σ plasmon is an intrinsic mode, driven by interband electronic transitions inherent to the material, and is relatively insensitive to external modulation. In comparison, the Dirac plasmon is an extrinsic mode, induced by external carrier doping, and exhibits high tunability. It is this extrinsic mode that constitutes the main focus of this study.

**Figure 3:**
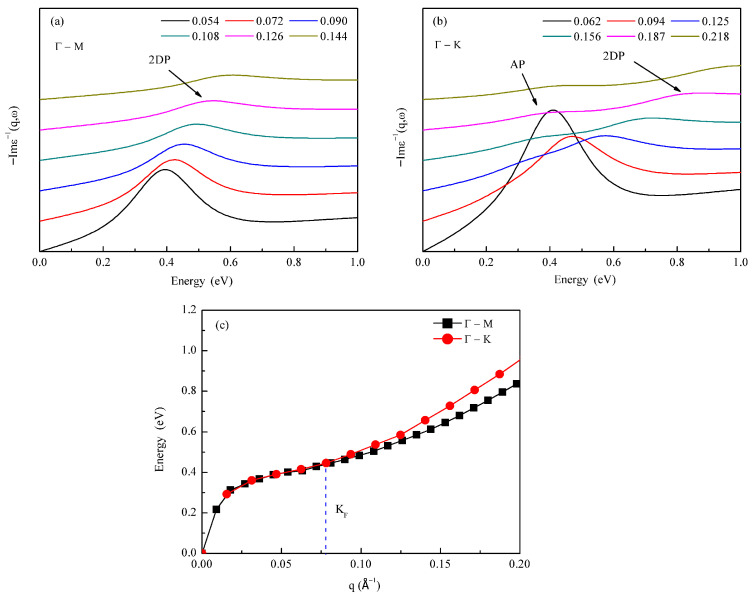
Directional anisotropy of dirac plasmons in germanene: (**a**) EELS spectra of doped germanene along the Γ–M direction with q ranging from 0.054 to 0.144 Å^−1^, showing a well-defined single-peak 2D Dirac plasmon (2DP) mode; (**b**) EELS spectra along the Γ–K direction (q = 0.062–0.218 Å^−1^), illustrating a transition from a single-peak to a double-peak structure beyond a critical q value; (**c**) the 2DP dispersion relations along Γ–M (black squares) and Γ–K (red circles). The blue dashed line marks the Fermi wave vector k_F_.

To gain deeper insight into the characteristics of Dirac plasmons in germanene, we analyzed the low-energy region (0–1 eV) of the electron energy loss spectroscopy (EELS) along different high-symmetry directions for a carrier-doped germanene system with a concentration of 0.05 electrons per unit cell. Figure 3a,b show the spectra along the Γ–M and Γ–K directions, respectively. Comparison reveals that the plasmon structure along the Γ–M direction is relatively simple, exhibiting a single peak corresponding to the conventional two-dimensional Dirac plasmon (2DP). In contrast, along the Γ–K direction, a distinct double-peak structure emerges at larger wave vectors (q ≥ 0.156 Å^−1^): the higher-energy peak is identified as the conventional 2DP, while the lower-energy peak is attributed to an acoustic plasmon (AP) [50]. The emergence of the acoustic plasmon originates from the coupling between charge carriers with different Fermi velocities [51]. As illustrated in Figure 1b, the reciprocal space paths help clarify this behavior: starting from the Dirac point K, only one type of propagation mode (K–K type, indicated by the blue arrow, corresponding to the short diagonal of the hexagonal Brillouin zone) exists along the Γ–M direction. In contrast, along the Γ–K direction, two distinct propagation paths are present: K–M (red arrow, parallel to the hexagon edge) and K–Γ (red arrow, along the diagonal direction). First-principles calculations reveal a slight anisotropy in the electronic structure of germanene near the K point: the Fermi velocity along the K–K path is approximately 5.19 × 10^5^ m/s, while those along the K–M and K–Γ paths are 5.06 × 10^5^ m/s and 5.36 × 10^5^ m/s, respectively. The coupling between carriers with these different Fermi velocities gives rise to the unique double-peak plasmon structure along the Γ–K direction. This study focuses primarily on the fundamental properties of the conventional two-dimensional Dirac plasmon (2DP).

Furthermore, we systematically analyzed the dispersion relations of the two-dimensional Dirac plasmon in germanene as a function of the wave vector q along the Γ–M and Γ–K directions, as shown in Figure 3c. The behavior can be divided into two regions, with a critical point at the Fermi wave vector k_F_: in the region where q < k_F_, plasmons cannot decay into energy-momentum-matched electron–hole pairs (single-particle excitations) and thus theoretically exhibit longer lifetimes, following a √q dispersion relation. For q > k_F_, Landau damping [52] sets in, allowing plasmons to dissipate energy by generating electron–hole pairs, leading to shortened lifetimes and a quasi-linear dispersion. Figure 3c also shows that in the non-Landau damping region (q < k_F_), the plasmon dispersions along both directions nearly coincide, indicating good isotropy. In the Landau damping region (q > k_F_), however, the plasmon energy along the Γ–K direction is slightly higher than that along the Γ–M direction. The slight anisotropy in plasmon energy between the Γ–K and Γ–M directions arises from a mode-splitting mechanism. At small wavevectors (q < k_F_), the plasmon dispersion is nearly isotropic, as seen in Figure 3c. For larger q (q > k_F_), beyond the onset of Landau damping, the plasmon branch along the Γ–K direction splits into an acoustic plasmon mode and a conventional two-dimensional Dirac plasmon (2DP) mode (Figure 3a,b). The energy repulsion between these two split modes pushes the 2DP to a higher energy, resulting in its slightly higher energy compared to the 2DP along the Γ–M direction at the same wavevector. We anticipate that this phenomenon could be experimentally observed using high-resolution angle-resolved electron energy loss spectroscopy (HREELS).

### 3.2. Carrier Concentration Modulation for Germanene Dirac Plasmons

**Figure 4:**
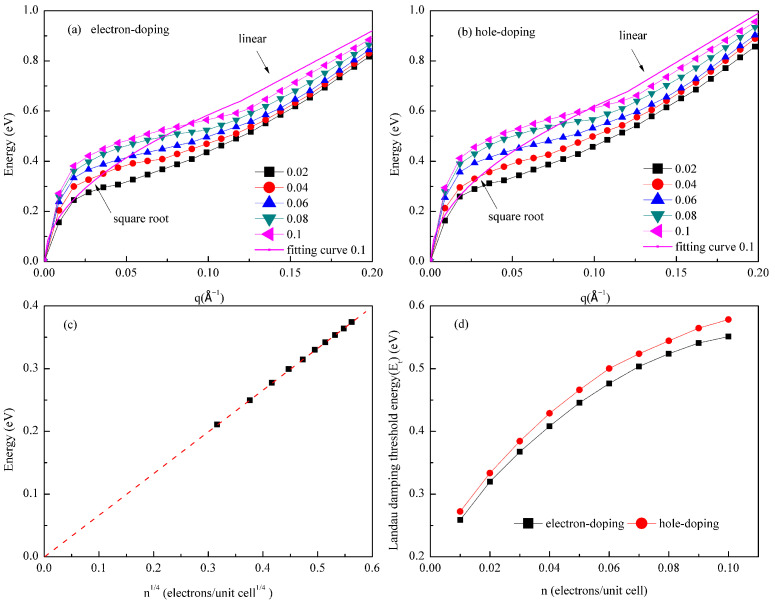
Modulation of Dirac plasmons in germanene by carrier doping: (**a**) Plasmon dispersion along the Γ–M direction in germanene under electron doping at various carrier concentrations n (in units of electrons per unit cell). For the case of *n* = 0.1, the dispersion relation is fitted with a piecewise function: a square-root function (ω ∝ √q) for q < k_F_ and a linear function for q > k_F_. The pink curve represents the combination of these two independent fitting segments, demonstrating that a single square-root function is inadequate for describing the dispersion across the entire q-range; (**b**) plasmon dispersion along the Γ–M direction in germanene under hole doping across multiple carrier concentrations, the pink fitted curve also represents the dispersion relation exhibits a √q dispersion relationship at smaller q values, while quasi-linear characteristics at larger q values at the carrier concentration of 0.1 electrons/unit cell; (**c**) scaling relation between the plasmon energy and carrier concentration (proportional to n^1/4^) at a fixed wave vector q = 0.018 Å^−1^; (**d**) Dirac plasmon threshold energy as a function of carrier concentration for both electron doping (black squares) and hole doping (red circles).

In experimental settings, doping can be achieved through chemical adsorption or gate voltage modulation. In this work, the carrier concentration is tuned by shifting the Fermi level (E_F_) within the framework of the rigid band approximation. An upward shift of E_F_ increases the number of occupied electronic states below it, resulting in n-type doping with a higher electron concentration. Conversely, a downward shift of E_F_ decreases the occupation of states, leading to p-type doping with an increased hole concentration. The extent of the Fermi level shift directly determines the doping level, enabling continuous and controlled carrier modulation. Figure 4 systematically investigates the effect of doping on the behavior of Dirac plasmons in germanene. Figure 4a,b show the dispersion relations of Dirac plasmons along the Γ–M direction under electron doping and hole doping, respectively, at various carrier concentrations. The results indicate that, regardless of doping type, the plasmon dispersion exhibits typical two-regime behavior at all concentrations: in the long-wavelength regime (small q), it follows the ω ∝ √q scaling relation, while in the short-wavelength regime (larger q), it transitions to quasi-linear behavior. As the absolute carrier concentration increases, the Fermi wave vector k_F_ also increases, shifting the critical point toward larger q values and inducing a systematic blueshift in the overall dispersion relation.

The observed √q dependence in the long-wavelength limit and the blueshift with increasing concentration can be explained by the dielectric theory of two-dimensional systems [53]. In this regime, plasmon behavior is governed by the polarizability Π(q, ω) and the two-dimensional Coulomb potential *v*(q). The polarizability is approximated as Π(q, ω) ≈ D_0_V_F_^2^q^2^(1 − ω^2^/4E_F_^2^)/2ω^2^, where D_0_ is the density of states at the Fermi level, V_F_ is the Fermi velocity, and *v*(q) = 2πe^2^/q. Within the random phase approximation (RPA), the dielectric function is given by ε(q, ω) = 1 − *v*(q)Π(q, ω), and the plasmon mode corresponds to the solution of ε(q, ω) = 0. From this, it can be deduced that the plasmon frequency satisfies.

(7) $$\omega =\sqrt{\pi e^{2}\gamma^{2}D_{0}}\sqrt{q}.$$

Therefore, it can be seen that the dispersion of two-dimensional Dirac plasmons follows ω ∝ √q in the long-wavelength limit. Furthermore, due to the linear band dispersion near the Dirac point in germanene, D_0_ ∝ E_F_/V_F_^2^, under the condition of a fixed q value, leading to ω∝√E_F_∝n^1/4^ relationship. This indicates that as the carrier concentration increases, the plasmon excitation energy increases, accounting for the observed blueshift. To validate this quantitative relationship, Figure 4c shows the plasmon energy as a function of n^1/4^ at q = 0.018/Å. The computational results (black squares) and linear fit (red dashed line) confirm a strict linear dependence within the concentration range of 0.01 to 0.1 electrons per unit cell.

Since the plasmon excitation energy varies with the wave vector, we use the energy at q = k_F_—referred to as the Landau damping threshold energy—as a representative quantity to conveniently and quantitatively characterize plasmon energy. This point lies at the boundary of the stable plasmon existence region (without Landau damping) and possesses clear physical significance. Figure 4d shows the variation in this threshold energy with carrier concentration under electron and hole dopings. As the carrier concentration increases from 0.01 to 0.1 per unit cell, the threshold energy rises from 0.26 eV to 0.55 eV (a 112% change) under electron doping, and from 0.27 eV to 0.58 eV (a 115% change) under hole doping. The slightly higher energy under hole doping stems from a mild asymmetry in the Dirac cone of germanene around the Fermi level: the valence band has a slightly higher slope than the conduction band, resulting in a larger shift in E_F_ for the same concentration under hole doping and hence a slightly higher excitation energy. Moreover, the threshold energy increases sublinearly with concentration, leading to a gradual decrease in tuning efficiency at higher concentrations. The plasmon excitation energy increases with carrier concentration, following the characteristic scaling law for Dirac plasmons, ω ∝ n^1/4^, as derived in Equation (7) and corroborated by the results in Figure 4c. This leads to a nonlinear increase in energy, with a diminishing tuning efficiency (dω/dn ∝ n^−3/4^) at higher densities. The underlying origin of this sublinear behavior lies in the conical band structure: at low carrier densities, the Fermi level is near the Dirac point, where the density of states is small, allowing for a significant energy shift with minimal added carriers. In contrast, at high densities, the same carrier addition produces a smaller energy change due to the larger density of states, resulting in the observed decrease in tuning efficiency.

### 3.3. Biaxial Strain Modulation for Germanene Dirac Plasmons

**Figure 5:**
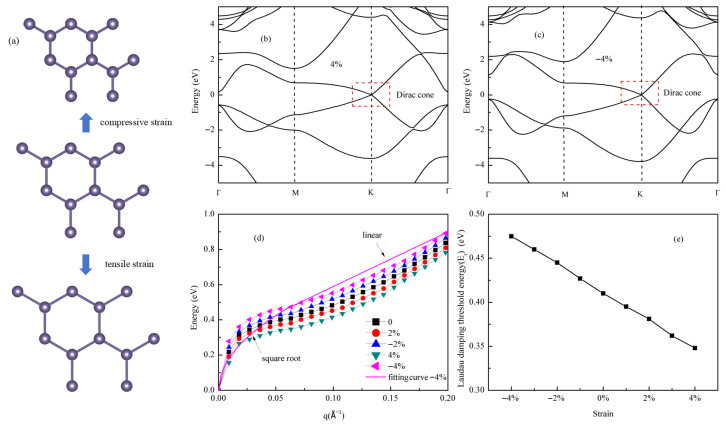
Modulation of Dirac plasmon properties in germanene via biaxial strain: (**a**) the schematic diagram of the germanene lattice under applied equibiaxial compressive and tensile strain; (**b**) band structure of germanene under 4% biaxial tensile strain; (**c**) band structure of germanene under 4% biaxial compressive strain; (**d**) plasmon dispersion along the Γ–M direction under varying biaxial strains (−4% to +4%) at a carrier concentration of *n* = 0.05 electrons/unitcell, the pink curve represents the fitted dispersion relation at a carrier concentration under 4% biaxial compressive strain, it exhibits a √q dispersion relationship at smaller q values, while quasi-linear characteristics at larger q values; (**e**) evolution of the Landau damping threshold energy for Dirac plasmons as a function of biaxial strain.

In practical applications, germanene is typically grown on substrates, which inevitably introduces lattice mismatch strain. Therefore, understanding the effect of strain on its physical properties is crucial. Uniaxial and biaxial strains affect germanene in fundamentally different ways [54]: uniaxial strain breaks the symmetry of the hexagonal lattice, causing the Dirac cone to split and opening a bandgap at the Dirac point. This originates from strain-induced inequivalence between the A and B sublattice sites. In contrast, equibiaxial strain preserves the lattice symmetry, shifting all Dirac points uniformly without opening a bandgap. According to relevant literature [55], for graphene, equibiaxial strain within a small range of ±5% maintains the linear dispersion of the Dirac cone and its semimetallic character. Figure 5a shows a schematic diagram of the germanene lattice under applied equibiaxial compressive and tensile strain, and the computational results in this study are consistent with previous work: as shown in Figure 5b,c, under both 4% (tensile) and −4% (compressive) equibiaxial strain, germanene retains the typical Dirac cone structure near the Fermi level at the Dirac point. This symmetry-protected band characteristic enables germanene to maintain its capability for Dirac plasmon excitation even under strain. For all strained germanene structures in this study, atomic position relaxation was performed. The corresponding buckling parameters are summarized in Table 1. The results indicate that when tensile strain is applied, the structure tends to become flatter, resulting in reduced buckling. In contrast, under compressive strain, the atoms release stress by arching upward, leading to increased buckling. These structural adaptations represent the system’s response to achieve a new energy-minimized state. This study also systematically investigates the effect of equibiaxial strain (ranging from −4% to 4%) on the plasmon dispersion of germanene at a fixed doping concentration of 0.05 electrons per unit cell, as shown in Figure 5d. The results indicate that the dispersion curves under all strain conditions still exhibit typical two-regime behavior: obeying the ω ∝ √q scaling law in the long-wavelength region and transitioning to a quasi-linear relationship in the short-wavelength region. Since the doping concentration is fixed, the Fermi wave vector k_F_ remains essentially unchanged, and thus the critical point position is consistent across strains. Notably, as the strain changes from compressive to tensile, the entire plasmon dispersion undergoes a systematic redshift.

Furthermore, to quantitatively evaluate the tuning capability of biaxial strain on the plasmon excitation energy, Figure 5e shows the variation in the threshold energy at q = k_F_ with strain. Over the strain range from −4% to 4%, the threshold energy changes linearly with strain: decreasing from 0.48 eV under compressive strain (−4%) to 0.35 eV under tensile strain (4%), representing a relative tuning range of 27.1%. These results confirm that the excitation energy of germanene plasmons can be effectively tuned within the range of 0.35–0.48 eV via biaxial strain.

**Figure 6:**
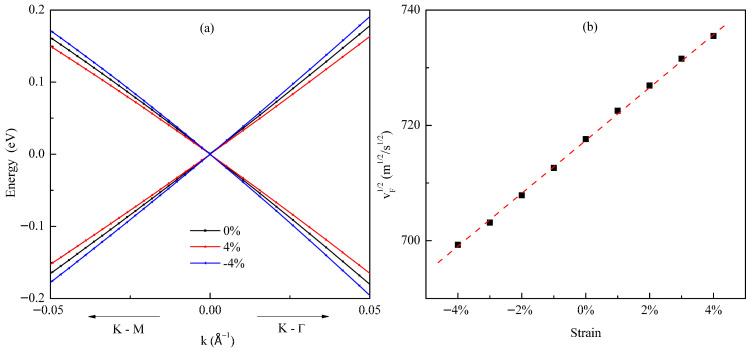
Mechanism of biaxial strain modulation on plasmon properties in germanene: (**a**) evolution of the electronic band structure near the K point under biaxial strain: compressive strain (–4%, blue), unstrained (0%, black), and tensile strain (+4%, red); (**b**) scaling relation between the strain and fermi velocity (proportional to v_F_^1/2^) at a carrier concentration of *n* = 0.05 electrons per unitcell.

The modulation of plasmon energy by strain is mediated through the strain-induced changes in the Fermi velocity (V_F_). As clearly shown in the band structures of Figure 6a, the slope of the Dirac cone decreases under tensile strain and increases under compressive strain, indicating a corresponding decrease or increase in V_F_. This change in V_F_ directly impacts the plasmon energy. According to the theoretical model for 2D Dirac plasmons, the plasmon frequency scales as ω ∝ E_F_^1/2^. Substituting the expression for the Fermi energy, E_F_ = ħV_F_k_F_, and considering a fixed carrier concentration (constant k_F_), this relationship simplifies to ω ∝ V_F_^1/2^. To confirm this mechanism, we plot the square root of the computed V_F_ (√V_F_) against the applied strain in Figure 6b. The resulting linear dependence provides direct quantitative evidence that the linear modulation of plasmon energy with strain, observed in Figure 5d, originates from the strain-controlled Fermi velocity via the ω ∝ V_F_^1/2^ scaling law.

### 3.4. Cooperative Modulation of Dirac Plasmon in Germanene by Carrier Concentration and Biaxial Strain

**Figure 7:**
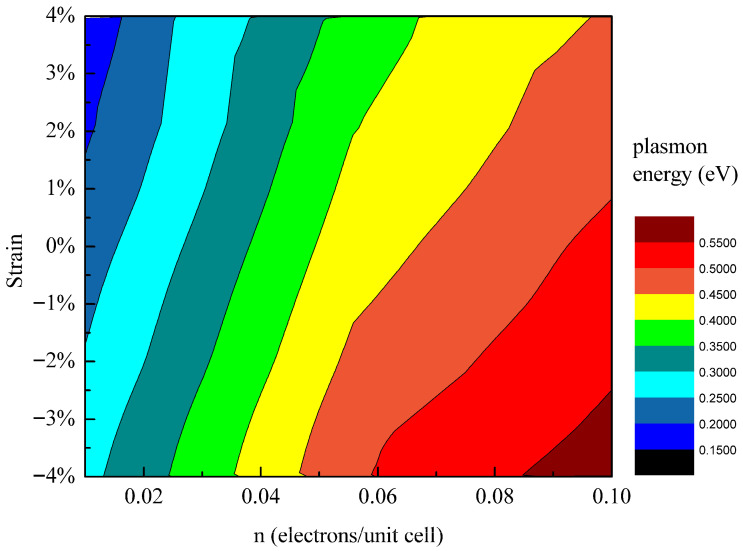
Two-dimensional phase diagram of the Landau damping threshold energy for germanene Dirac plasmons. The plasmon energy (color bar) is plotted as a function of carrier concentration (horizontal axis) and biaxial strain (vertical axis).

Based on the systematic investigations of the individual modulation effects of carrier doping (Section 3.2) and biaxial strain (Section 3.3) on germanene Dirac plasmons, both parameters have been demonstrated as efficient and precise tuning knobs. However, in practical device scenarios, carrier doping and lattice strain often coexist and may exhibit coupled synergistic effects. To go beyond single-parameter tuning and provide a comprehensive design blueprint for future multifunctional devices, we systematically mapped the Landau damping threshold energy of germanene Dirac plasmons as a function of both carrier concentration and biaxial strain, as shown in Figure 7.

This two-dimensional phase diagram clearly reveals a characteristic distribution: the plasmon energy is lower in the upper-left region (high tensile strain and low doping) and higher in the lower-right region (high compressive strain and high doping), consistently confirming the individual trends identified in previous sections. More importantly, the synergistic control strategy significantly expands the tunable range of the plasmon energy. Within the parameter space studied (carrier density from 0.01 to 0.1 electrons per unit cell and strain from −4% to +4%), the plasmon energy can be modulated widely from 0.16 eV to 0.61 eV, a range substantially broader than that achievable by varying only one parameter.

As revealed in Section 3.2 and Section 3.3, the plasmon energy follows the scaling relations ω ∝ n^1/4^ (carrier concentration dependence) and ω ∝ V_F_^1/2^ (Fermi velocity dependence). This behavior can be intuitively understood through a spring–mass oscillator model, where the plasmon collective oscillations are analogous to a classical oscillating system with frequency given by ω = √(k/m). Within this picture, variations in carrier concentration primarily affect the effective spring constant k—an increase in concentration enhances the Coulomb restoring force, thereby stiffening the system and raising the plasmon energy. In contrast, strain modulates the Fermi velocity by altering the slope of the Dirac cone, which in turn influences the effective mass m of the charge carriers—tensile strain flattens the energy bands, increasing the effective mass and consequently reducing the oscillation frequency. This model clearly elucidates the distinct physical origins of the two tuning mechanisms: carrier doping modifies the restoring characteristics of the system, while strain tailors the inertial response of the carriers. Together, they provide a complementary approach for achieving broad-range tunability of plasmon energy.

### 3.5. The Effect of Substrate on Dirac Plasmons in Germanene

**Figure 8:**
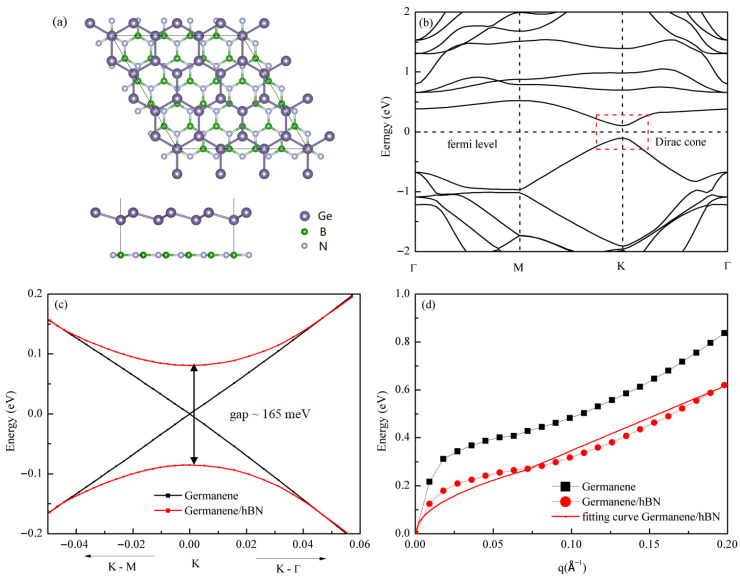
Substrate effects on the Dirac plasmon in germanene: (**a**) top and side views of the 3 × 3 germanene/5 × 5 hexagonal boron nitride (hBN) heterostructure; (**b**) band structure of the heterostructure near the Fermi level, showing a bandgap opening near the K point due to substrate hybridization. The Dirac cone region is highlighted by a red dashed box; (**c**) comparison of the band structures near the K point for pristine germanene (black curve) and the germanene/hBN heterostructure (red curve), revealing a bandgap difference of approximately 165 meV; (**d**) comparison of the Dirac plasmon dispersion relations along the Γ–M direction between pristine germanene and germanene/hBN at a doping concentration of 0.05 electrons/unitcell. The red curve represents the fitted dispersion relation for germanene/hBN, which exhibits a √q dependence at small q values and quasi-linear characteristics at larger q values.

While freestanding germanene monolayers are challenging to realize in practice and require substrate-assisted synthesis, metallic substrates typically disrupt the Dirac cone characteristics through strong interfacial orbital hybridization. In contrast, wide-bandgap semiconductor substrates such as hexagonal boron nitride (hBN) can help preserve these electronic properties [56]. In this study, we employed hBN as the substrate. To address the lattice mismatch between hBN (2.50 Å) and germanene (4.06 Å), we constructed a 5 × 5 hBN/3 × 3 germanene supercell, as shown in Figure 8a, which reduces the lattice mismatch to 2.4%. Furthermore, structural optimization using the DFT-D2 method [57] yielded a supercell lattice constant of 12.31 Å and an interlayer distance of approximately 3.56 Å.

Figure 8b presents the band structure of this heterostructure. Although considerably more complex than that of pristine germanene, Dirac cone-like features persist near the K point above and below the Fermi level, with a small bandgap opening due to substrate interaction. A comparative analysis of the band structures near the K point between the heterostructure and isolated germanene, as shown in Figure 8c, reveals that at larger wave vectors (|k| > ~0.04 Å^−1^), the bands nearly coincide with those of pristine germanene, exhibiting a near-linear relationship. However, at smaller momenta close to the K point (|k| < ~0.04 Å^−1^), the dispersion becomes parabolic, opening a bandgap of approximately 165 meV. This raises an important question: can such a hybrid band structure (linear at larger k, quadratic near K) maintain the √q dispersion relation observed in pristine germanene?

Theoretically, this system resembles a two-dimensional free electron gas (2DEG) model with a parabolic band structure. According to the dielectric theory of 2DEG, plasmons occur when the dielectric function satisfies ε(q, ω) = 1 − v_c_(q)Π(q, ω) = 0. Here, however, the polarizability takes the form Π(q, ω) ≈ (nq^2^)/(m*ω^2^), where m* represents the effective band mass. Solving this equation yields ω = √[(2πe^2^n)/(κm*)]√q, indicating that systems with parabolic bands can still maintain the √q dispersion relation for plasmons. Therefore, theory predicts that the germanene heterostructure should preserve the √q plasmon dispersion.

This prediction is confirmed in Figure 8d. Through detailed time-dependent density functional theory within the random phase approximation (TDDFT-RPA) calculations, we find that although the electronic structure is modified, the resulting plasmon dispersion preserves the characteristic √q dependence at small wave vectors and transitions into a nearly linear dispersion at larger wave vectors. This behavior aligns with previously reported characteristics of bilayer graphene [58], suggesting that analogous band structures yield similar dispersion outcomes. Furthermore, the dispersion closely resembles that of freestanding germanene, albeit with a noticeable energy redshift. These results indicate that the introduction of a substrate does not alter the fundamental qualitative nature of the plasmon dispersion.

## 4. Conclusions

In summary, this work systematically investigates the modulation of Dirac plasmons in germanene through carrier doping, biaxial strain, and substrate effects using first-principles LR-TDDFT-RPA calculations. The Dirac plasmons in germanene exhibit significant directional anisotropy: only a single 2DP mode appears along the Γ–M direction, while an additional acoustic plasmon emerges along the Γ–K direction due to coupling between carriers with slightly different Fermi velocities. We establish the quantitative scaling relation ω ∝ n^1/4^ for carrier concentration dependence, which leads to a sublinear increase in plasmon energy with doping level. Furthermore, we derive and validate the fundamental relationship ω ∝ √V_F_ that directly explains the linear modulation of plasmon energy by strain. The synergistic control between carrier density and strain significantly expands the tunable range of plasmon energy from 0.16 eV to 0.61 eV, substantially broader than what can be achieved by varying only one parameter. When supported on hBN substrates, germanene maintains the characteristic √q plasmon dispersion despite substantial band structure modification and a noticeable redshift in plasmon energy. This behavior is well captured by the random phase approximation applied to a two-dimensional electron system with parabolic band dispersion, where the long-range Coulomb interaction remains essential for sustaining the collective plasmon mode. These findings provide comprehensive insights into the active tuning mechanisms of Dirac plasmons in germanene and establish a solid theoretical foundation for developing germanene-based tunable nanophotonic devices.

## Figures and Tables

**Table 1:** Buckling parameters of germanene as a function of biaxial strain.

Strain	−4%	−3%	−2%	−1%	1%	2%	3%	4%
Bulking Parameters (Å)	0.790	0.756	0.738	0.702	0.661	0.648	0.636	0.624

**Figure A1:**
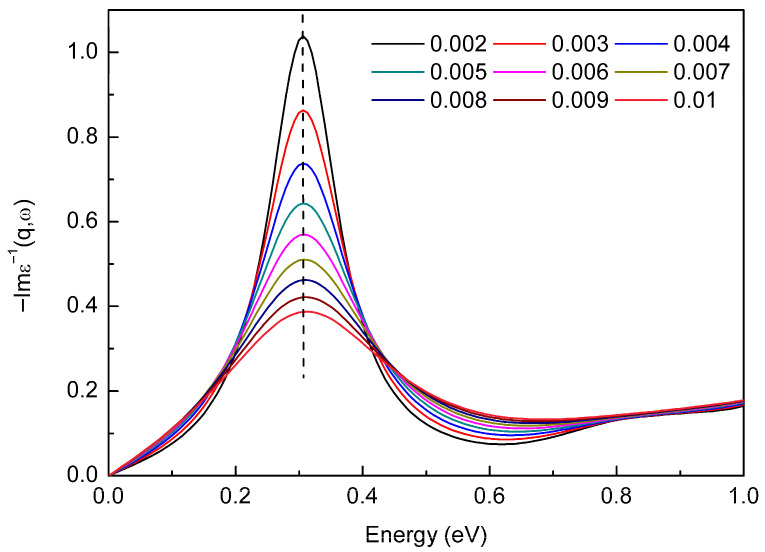
Momentum-resolved electron energy loss spectroscopy (EELS) of germanene along the Γ–M direction at momentum transfer q = 0.018 Å^−1^ with the broadening parameter η from 0.002 Ry to 0.01 Ry. The position of the corresponding plasmon peak remains essentially unchanged with variations in the broadening parameter η.

## Data Availability

The original contributions presented in this study are included in the article. Further inquiries can be directed to the corresponding author.

## Appendix A

To verify the appropriateness of the broadening parameter (η = 0.01 Ry) used in this work, we systematically examined its impact on the electron energy loss spectrum (EELS). As shown in Figure A1, calculations with η values ranging from 0.002 Ry to 0.01 Ry confirm that while the peak width broadens as η increases, the position of the plasmon peak remains virtually unchanged. Since the central objective of our analysis is the accurate determination of the plasmon energy (i.e., the peak position), the chosen value of η = 0.01 Ry is well justified and does not artificially shift the key quantitative results of this study.
